# Case Report: Partial Splenic Artery Embolization for the Treatment of Painful Splenomegaly Secondary to Noncirrhotic Portal Hypertension

**DOI:** 10.1155/crhe/9303407

**Published:** 2025-06-06

**Authors:** Logan Kratzer, Maddison Waters, Simon Parkes, Nicholas Cheung, Nicholas Shackel

**Affiliations:** ^1^Department of Gastroenterology, Launceston General Hospital, Launceston, Tasmania, Australia; ^2^Tasmanian School of Medicine, University of Tasmania, Launceston, Tasmania, Australia; ^3^Medical Research Department, Clifford Craig Foundation, Launceston, Tasmania, Australia

**Keywords:** noncirrhotic portal hypertension, painful splenomegaly, partial splenic artery embolization

## Abstract

Noncirrhotic portal hypertension (NCPH) is a rare cause of portal hypertension with varied etiologies. We present two cases of painful splenomegaly secondary to NCPH successfully treated with partial splenic artery embolization (PSE). Despite limited literature on PSE's efficacy for NCPH–related painful splenomegaly, our cases demonstrate significant pain relief and reduction in opiate dependence postprocedure. Imaging revealed substantial decreases in spleen size without major complications. PSE emerges as a promising therapeutic option for NCPH–related painful splenomegaly, offering improved quality of life and reduced reliance on analgesics.

## 1. Introduction

Noncirrhotic portal hypertension (NCPH) stands as a distinct, noncirrhosis-related cause of portal hypertension. In the Western World, its incidence is between 3% and 5% [1]. In developed regions, etiologies include childhood infections, immunological disorders, medications, toxins, genetic disorders, and thrombophilias [2]. Common clinical presentations include gastrointestinal hemorrhage, cytopenias related to splenomegaly, and less commonly, painful splenomegaly [3]. Notably, the prognosis for NCPH is more favorable compared to cirrhosis-related portal hypertension [3]. Management of varices involves regular screening gastroscopy and band ligation. While the management of symptomatic splenomegaly is not as well described, it generally involves either surgical splenectomy or partial splenic artery embolization (PSE) [4]. PSE has shown promising results for improving hematological parameters, as well as the risk of variceal hemorrhage [4, 5], and is well tolerated [6, 7]. However, few cases have explored its impact on alleviating painful splenomegaly [8]. In this report, we present 2 cases in a regional hospital in Australia, in which PSE resulted in significant relief from painful splenomegaly.

## 2. Case Report

### 2.1. Case 1

A 40-year-old female was referred to the hepatology clinic for chronic left upper quadrant abdominal pain, attributed to NCPH–related splenomegaly, secondary to a portal vein thrombosis (PVT) from perinatal insult diagnosed at age 14. The patient had two previous episodes of variceal hemorrhage during her adolescence, which were managed through variceal banding. Her treatment for PVT had included anticoagulation with warfarin, which was later discontinued due to evidence of PVT recanalization on serial imaging and normal thrombophilia testing.

Previously, the management of her painful splenomegaly involved high doses of oral opiates, up to 100 mg morphine equivalent per day, a spinal cord stimulator, and nine hospital admissions for ketamine infusions. Despite these interventions, her pain remained poorly controlled, significantly negatively impacting her quality of life.

**Figure 1:**
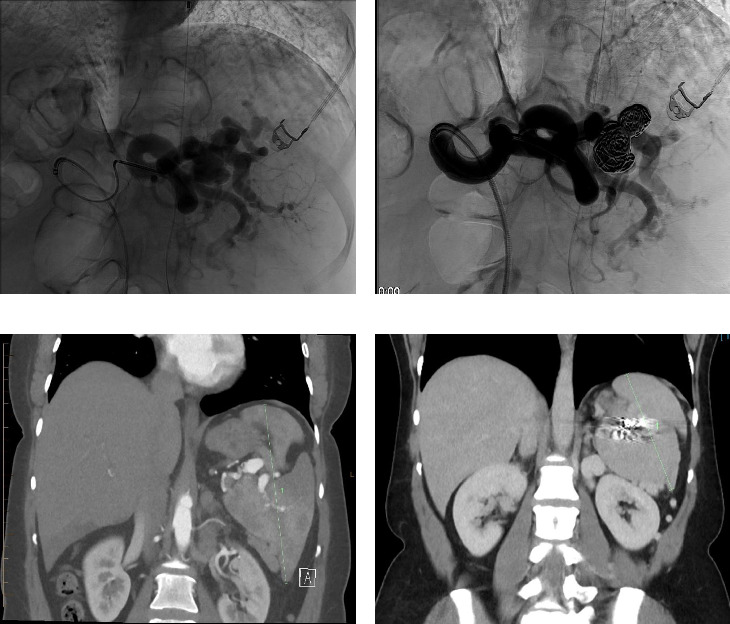
Splenic artery embolization with coil embolization for the treatment of painful splenomegaly secondary to extrahepatic portal hypertension. (a) DSA of splenic artery aneurysm prior to coil embolization. (b) Splenic artery aneurysm postcoil and glue embolization. (c) Craniocaudal splenic measurement pre-embolization, 158 mm. (d) Craniocaudal splenic measurement postembolization, 88 mm.

Initial assessment with computerized tomography (CT) demonstrated splenomegaly measuring 158 mm in the craniocaudal dimension, two splenic artery aneurysms, the largest measuring 21 × 15 mm, and multiple splenorenal varices (Figure 1). Given the absence of a physical shunt or significant intraabdominal collaterals, a surgical shunt was not considered. Instead, the patient underwent PSE with insertion of coils and lipoidal with the anticipated outcome being a reduction in splenic volume and improvement in pain.

The procedure was complicated by postembolization syndrome, resulting in an acute worsening of pain and nausea, necessitating a nine-day hospital admission for symptom management. In the follow-up CT scan five months postprocedure, there was a 70% reduction in blood flow and a decrease in spleen size to 88 mm in the craniocaudal dimension. During the initial three-month review, the patient had complete resolution of her pain with no further requirement for opiates. This benefit has persisted during subsequent follow-up assessments at 15 months.

### 2.2. Case 2

A 54-year-old female with NCPH, due to pregnancy-related PVT at the age of 39, was referred to the hepatology clinic for management of worsening painful splenomegaly.

The patient's NCPH was complicated by painful splenomegaly, Grade 1 esophageal varices without complicating hemorrhage, and no cytopenias. During subsequent workup, the patient was found to be JAK2 positive and confirmed to have myeloproliferative neoplasm favoring myelofibrosis on a bone marrow biopsy.

Previous treatment for the NCPH had included warfarin for 2 years. This was subsequently ceased due to labile INR and patient preference. Patient's pain was managed with regular oral opiates and one hospital admission for acute pain crisis. The pain was reported as severe on most days limiting the patient's ability to carry out her activities of daily living and work.

**Figure 2:**
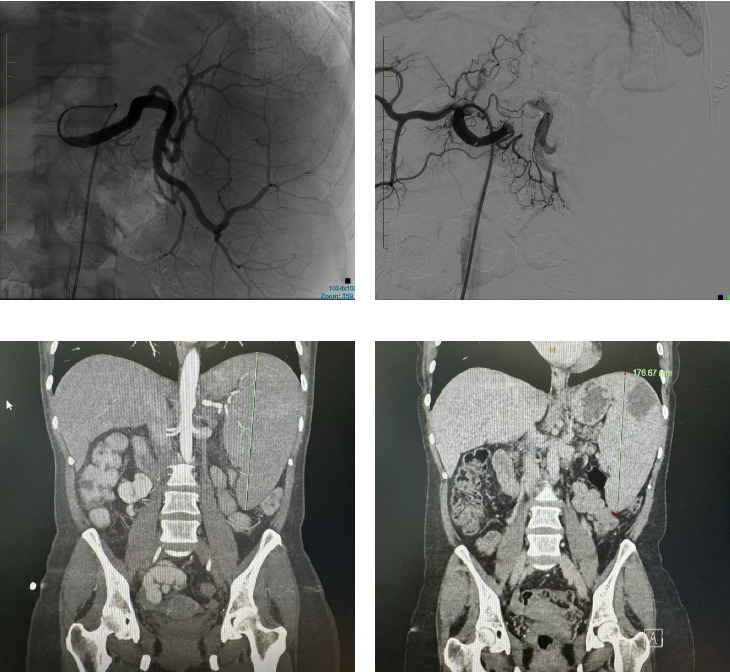
Splenic artery embolization with the Amplatzer plug for the treatment of painful splenomegaly secondary to extrahepatic portal hypertension. (a) DSA of splenic artery aneurysm prior to Amplatzer plug embolization. (b) Splenic artery aneurysm post-Amplatzer plug embolization. (c) Craniocaudal spleen measurement pre-embolization, 203 mm. (d) Craniocaudal splenic measurement postembolization, 176 mm.

This patient underwent PSE with a 12 mm Amplatzer plug into the midsplenic artery. The procedure was uncomplicated, and the patient was discharged at 24 h postprocedure. On the initial 3-month review, the splenic diameter had reduced from a maximal craniocaudal dimension of 203 mm to 176 mm, with multiple small areas of splenic infarction (Figure 2). The patient had a complete resolution of her chronic pain syndrome with no further reliance on oral analgesia.

For the management of her myeloproliferative neoplasm, the patient was commenced on aspirin to prevent thrombotic complications. In the absence of cytopenias or constitutional symptoms, cytoreductive therapy was not commenced.

## 3. Discussion

In this report, we present two cases of painful splenomegaly secondary to NCPH, both successfully treated with PSE. While PSE has been previously described in the literature for the treatment of variceal hemorrhage and hematological abnormalities in splenomegaly, limited outcomes have been reported regarding its efficacy in improving the symptoms of painful splenomegaly. Our findings suggest that PSE represents a viable treatment option for patients with NCPH suffering from painful splenomegaly, owing to its low rates of complications and the subsequent reduction in opiate dependence.

## Data Availability

The data supporting the findings of this case report are not publicly available due to patient privacy and confidentiality concerns. De-identified data may be made available from the corresponding author upon reasonable request and with appropriate institutional and ethical approvals.

## References

[1] Schouten J. N., Garcia-Pagan J. C., Valla D. C., Janssen H. L. (2011). Idiopathic Noncirrhotic Portal Hypertension. *Hepatology (Baltimore, Md.)*.

[2] Khanna R., Sarin S. K. (2018). Idiopathic Portal Hypertension and Extrahepatic Portal Venous Obstruction. *Hepatology International*.

[3] Schouten J. N., Verheij J., Seijo S. (2015). Idiopathic Non-Cirrhotic Portal Hypertension: A Review. *Orphanet Journal of Rare Diseases*.

[4] Ozturk O., Eldem G., Peynircioglu B. (2016). Outcomes of Partial Splenic Embolization in Patients With Massive Splenomegaly Due to Idiopathic Portal Hypertension. *World Journal of Gastroenterology*.

[5] Koconis K. G., Singh H., Soares G. (2007). Partial Splenic Embolization in the Treatment of Patients With Portal Hypertension: A Review of the English Language Literature. *Journal of Vascular and Interventional Radiology*.

[6] Gu J.-J., He X.-H., Li W.-T. (2012). Safety and Efficacy of Splenic Artery Coil Embolization for Hypersplenism in Liver Cirrhosis. *Acta Radiologica*.

[7] Hadduck T. A., McWilliams J. P. (2014). Partial Splenic Artery Embolization in Cirrhotic Patients. *World Journal of Radiology*.

[8] Grassi C. J., Boxt L. M., Bettmann M. A. (1987). Partial Splenic Embolization for Painful Splenomegaly. *CardioVascular and Interventional Radiology*.

